# Digital Implant Planning in Patients with Ectodermal Dysplasia: Clinical Report

**DOI:** 10.3390/ijerph19031489

**Published:** 2022-01-28

**Authors:** Lauren Bohner, Shankeeth Vinayahalingam, Johannes Kleinheinz, Marcel Hanisch

**Affiliations:** 1 Department of Oral and Maxillofacial Surgery, Hospital University Münster, 48149 Münster, Germany; johannes.kleinheinz@ukmuenster.de (J.K.); marcel.hanisch@ukmuenster.de (M.H.); 2 Department of Oral and Maxillofacial Surgery, Radboud University Medical Centre, 6525 Nijmegen, The Netherlands; shankeeth.vinayahalingam@radboudumc.nl

**Keywords:** dental implants, surgery, computer-assisted, rare diseases

## Abstract

Ectodermal dysplasia may severely affect the development of jaw growth and facial appearance. This case report describes the treatment of two patients suffering from ectodermal dysplasia, both treated with dental implant-fixed restorations by means of computer-guided surgery. Two patients presented to our clinic with congenital malformation of the jaw as a manifestation of ectodermal dysplasia, showing oligodontia and alveolar ridge deficit. Clinical examination revealed multiple unattached teeth and a need for prosthetic therapy. For both cases, dental implants were placed based on a computer-guided planning. A surgical guide was used to determine the positioning of the dental implants according to the prosthetic planning, which allowed for a satisfactory aesthetic and functional outcome. Computer-guided implant placement allowed predictable treatment of complex cases with satisfactory aesthetic and functional results. Adequate surgical and prosthetic planning is considered critical for treatment success.

## 1. Introduction

Ectodermal dysplasia may severely affect the development of jaw growth and facial appearance. This congenital condition affects ectodermal tissues, as hair, teeth, nails and sebaceous glands. In this regard, oral manifestations can vary from morphological anomalies to dental agenesis, which may be accompanied by bone atrophy and abnormal jaw relationship. Due to the multiple manifestations, it may negatively affect functional, aesthetic, and social aspects [1,2].

Prosthetic rehabilitation in patients with ectodermal dysplasia requires an accurate and personalized treatment plan. Treatment may include removable, fixed, or implant-supported prostheses, according to the severity of dental manifestations [3,4,5,6,7,8].

However, insertion of dental implants in jaws with bone atrophy may be challenging. To date, digital tools can be used as a support for predictable dental implant planning. Patients’ digital data are used as a reference to determine both future prosthetic rehabilitation and required treatments [9,10,11].

A computer-guided surgery may benefit dental implant rehabilitation of patients with ectodermal dysplasia, thus avoiding the risk of complication and injury of anatomical structures. This case report describes the treatment of two patients suffering from ectodermal dysplasia, both treated with dental implant-fixed restorations by means of computer-guided surgery.

## 2. Case Report

### 2.1. Case One

A 15-year-old patient attended the Dental Clinic of Hospital University Münster with aesthetic and functional complaint. Medical history and anamnesis revealed a diagnosis of Bloch–Siemens–Syndrome, Incontinentia pigmenti, and Epilepsy, which was previously confirmed by genetic testing. At the time of the consultation, the patient presented good health conditions. Extra-oral examination showed inadequate jaw relationship and loss of vertical dimension. Intra-oral and radiological examinations showed permanent tooth agenesis, and permanence of primary dentition, such as abnormally shaped teeth.

The interdisciplinary treatment plan included a previous orthodontic treatment, placement of dental implants after completion of mandibular growth and further rehabilitation with teeth-supported and implant-supported prosthesis. The complete oral rehabilitation of this patient was previously described in the literature [12].

**Figure 1:**
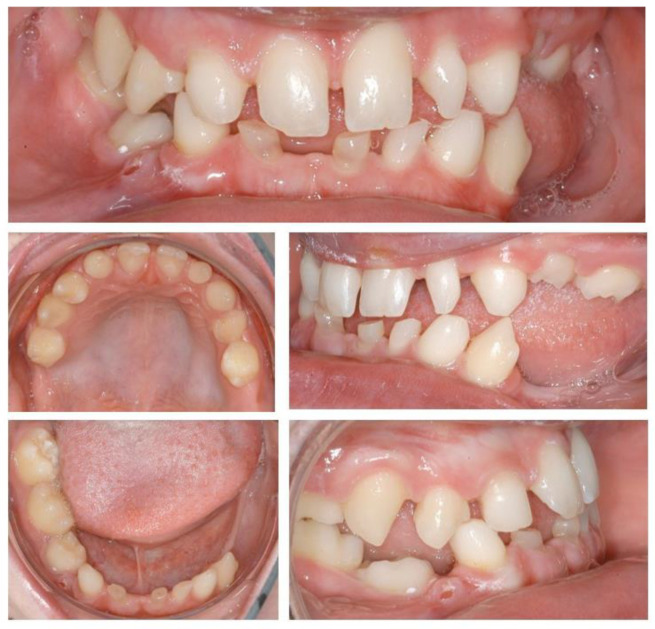
Initial situation. Clinical examination showed absence of permanent teeth and persistence of primary dentition, abnormally shaped teeth, and unsatisfactory jaw relationship, suggesting the need of interdisciplinary treatment.

**Figure 2:**
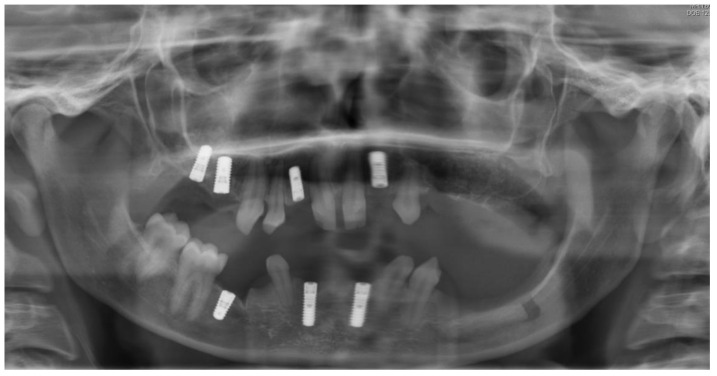
Panoramic radiography after bone graft, nerve lateralization, and placement of dental implants in edentulous sites with adequate bone volume.

As soon as the orthodontic treatment was completed, the patient returned for dental implant planning. Regardless of adequate teeth alignment, the wide interproximal spaces, disproportional teeth dimensions, and abnormal occlusion increased the complexity of the case (Figure 1). Radiological examinations showed a reduced bone height at the edentulous site. In consideration of a bone atrophy in the upper and lower jaws, a two-stage surgical procedure was planned, which included bilateral sinus lift, nerve lateralization and placement of dental implants at the sites 16, 15, 12, 22, 35, 36, 32, 42, and 45 (Figure 2).

The first surgical procedure included nerve lateralization at the left lower jaw, sinus lift, and placement of dental implants in edentulous sites with adequate bone volume. In order to reduce the risk of nerve injury related to the exacerbated bone atrophy at left lower jaw, the placement of dental implants at the left site was planned digitally (Figure 2).

**Figure 3:**
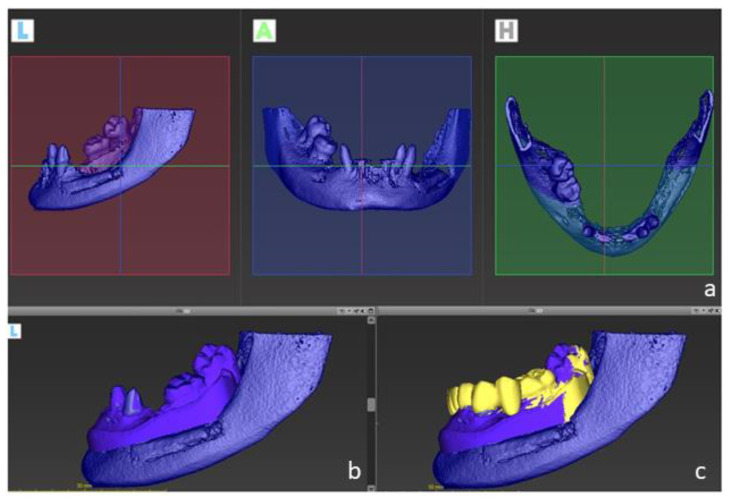
Digital files were taken to the virtual planning using an implant planning software. DICOM files (**a**) were matched to dental casts (**b**) and to the digital wax-up (**c**) to determine the optimal implant position.

**Figure 4:**
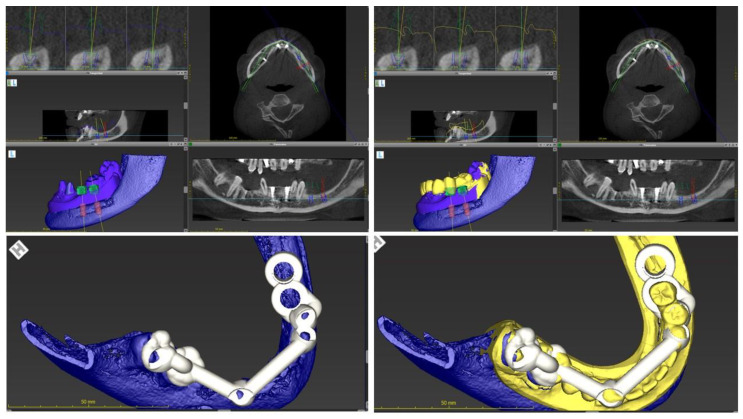
Based on anatomical and prosthetic references, a surgical guide was designed digitally and printed to be used as a reference during the surgical procedure. The figure left shows the surgical guide superimposed to the actual intra-oral situation. The figure right shows the surgical guide related with the designed wax-up.

The digital planning is shown in Figure 3 and Figure 4. A cone-beam computed tomography (CBCT) image was taken from the lower jaw using a CBCT device (Kavo, Kloten, Switzerland). Intra-oral scans were taken using a three-dimensional (3D) scanner (3Shape). Digital planning was conducted by a dental technician using the software Co-DiagnostiX (Dental Wings, Montreal, QC, Canada).

In summary, the “digital imaging and communications in medicine “(DICOM) files were imported to the software, and segmented automatically to comprise the region of interest. Next, “standard tessellation language” (STL) files were imported and superimposed upon DICOM-Data based on common points, which were defined by the user. In addition, the digital wax-up was matched to these files. This approach enabled to plan the implant location, taking into account the bone anatomy and the planned restorations.

Dental implants with appropriate dimensions (size and length) were selected from the library offered by the software to simulate the implant position. In order to avoid inaccuracies caused by dental implants-based artifacts, STL-Data were used as reference to determine the teeth surface. A surgical guide was designed based on the digital planning and printed using a 3D-printer (Formlabs) to guide the surgery.

**Figure 5:**
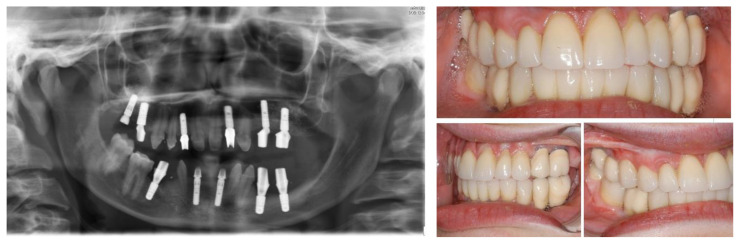
Left: final orthopantomogram showing the dental implants. Right: intra-oral situation after final rehabilitation with dental-supported and implant-supported prosthesis [12].

The placement of two titanium dental implants (Bone Level 4.1 × 8 mm, Straumann) at premolar and molar sites (35, 36) was planned. Surgical procedures were conducted under local anesthesia following a healing period of three months prior to implant exposure and prosthetic rehabilitation. Figure 5 shows the final prosthodontic rehabilitation at one year follow-up. Clinical outcomes showed osseointegration of dental implants and health of peri-implant tissues.

### 2.2. Case 2

A 25-year-old patient attended the Dental Clinic of Hospital University Münster about the unattractive smile and limited function. Anamnesis revealed an ectodermal dysplasia, which was confirmed genetically. No history of medication or allergy was given. Intraoral and radiological examination showed a permanent tooth agenesis, permanence of primary dentition and reduced vertical dimension. The interdisciplinary treatment included presurgical orthodontic treatment for teeth alignment and increase in vertical dimension, coronoplasty of anterior teeth, followed by implant placement at the sites 13, 23, and 25 and rehabilitation with implant-supported crowns.

**Figure 6:**
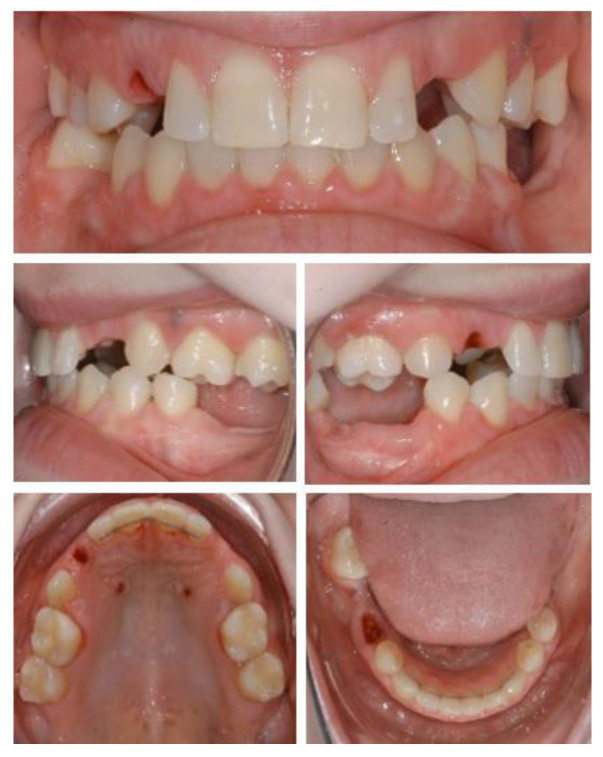
Intraoral situation after orthodontic treatment and extraction of deciduous teeth.

**Figure 7:**
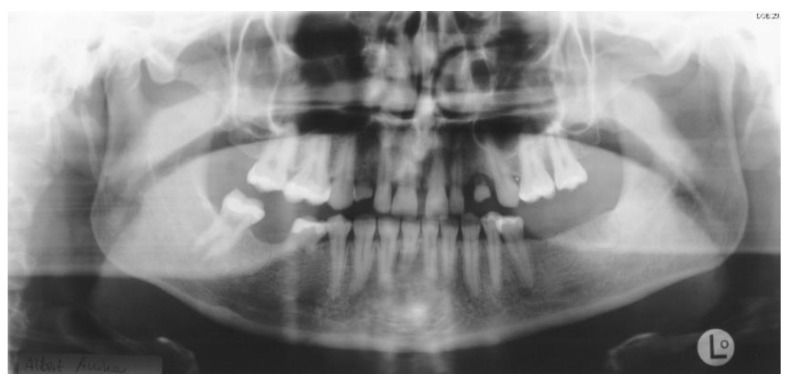
Initial panoramic radiography.

At the time of implant planning, orthodontic treatment was already finalized. Dental implants were planned to replace missing teeth at sites 13, 23, 36, 45, 46. Despite adequate teeth alignment, wide interproximal spaces at sites 13 and 23 revealed the need of combined restorative and surgical treatments (Figure 6 and Figure 7). Thus, dental implantation in the upper jaws was associated with enlargement of lateral incisors and correction of form in the case of teeth 13, 23.

**Figure 8:**
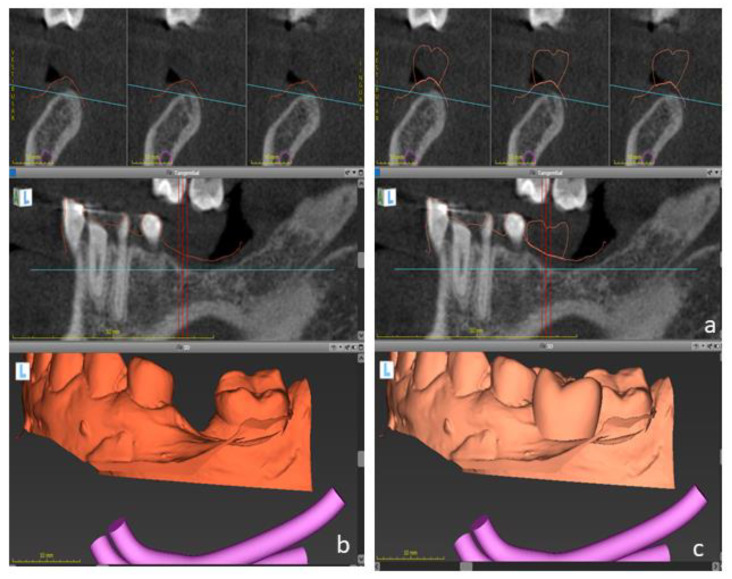
Dental implant planning on lower jaw. Sagittal cuts of cone-beam computed tomography (DICOM files) are shown (**a**). DICOM files were superimposed to the intraoral casts (**b**) and to the digital wax-up (**c**) to determine the optimal implant position.

**Figure 9:**
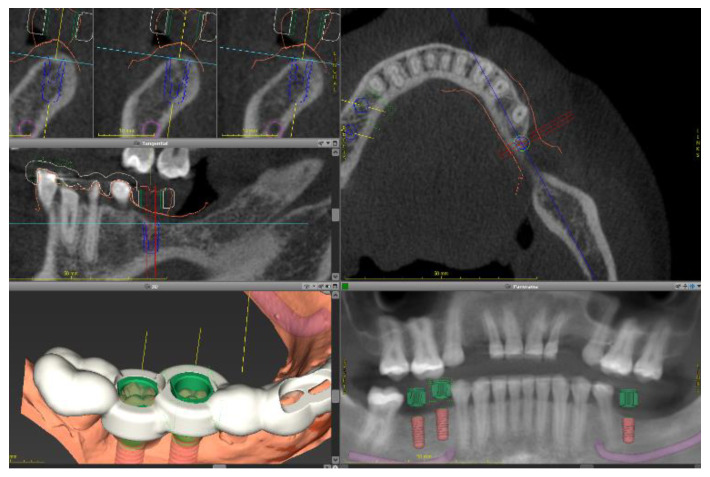
Design of a surgical guide to be used as a reference to dental implant placement.

Due to aesthetic concerns, the surgical procedure was planned digitally. CBCT imaging of both jaws was acquired with a CBCT device (Kavo). The examination showed sufficient bone volume on edentulous spaces. Thus, no bone or soft tissue graft procedures were required. A dental impression was taken with alginate and scanned with a desktop scanner (3Shape). In addition, a wax-up was designed digitally. Dental implant planning was conducted with the software Co-DiagnostiX, as described above (Figure 8). The CBCT images were matched to the intraoral digital casts and to the digital wax-up. On the basis of the implant planning, a surgical guide was designed and printed (Figure 9).

**Figure 10:**
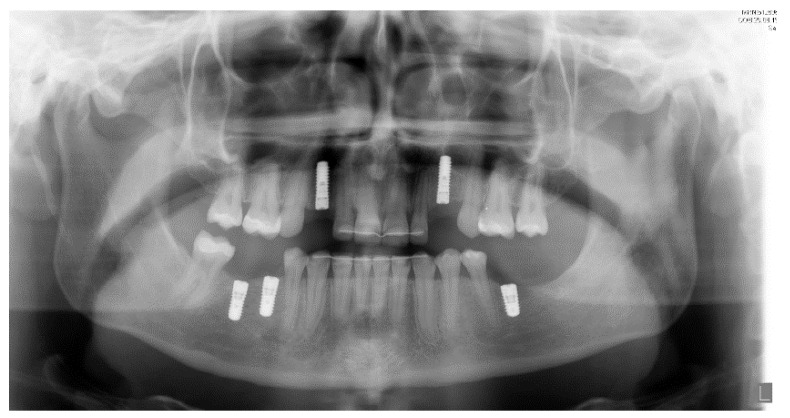
Post-operative panoramic radiography.

**Figure 11:**
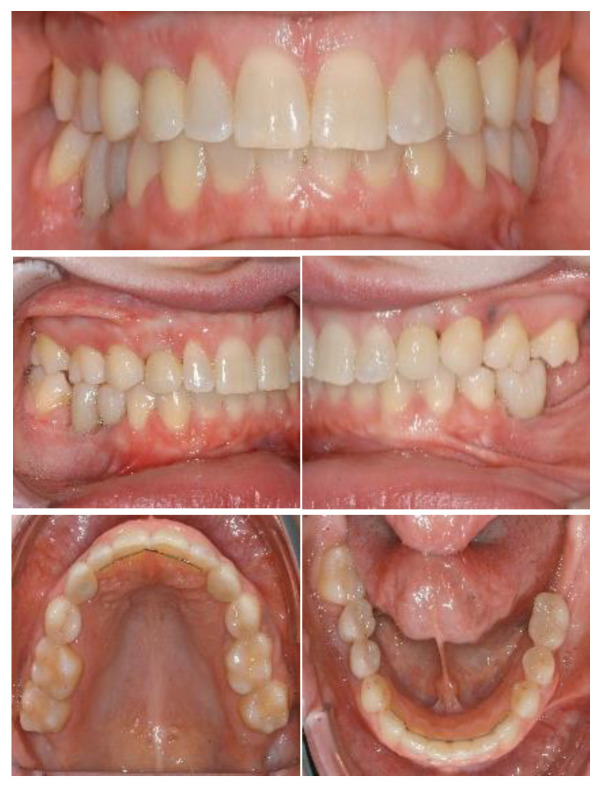
Definitive rehabilitation with dental-implant supported prosthesis.

Titanium dental implants (Bone Level, 3.33 × 12 mm and 4.1 × 8–10 mm, Straumann, Basel, Switzerland) were placed under local anesthesia (Septanest 1:200,000) at sites 13, 23, 36, 45, 46, using the surgical guide as reference. After a healing period of three months, implant-supported porcelain crowns were installed with a torque strength of 35 Ncm. Additionally, teeth 13 and 23 were prepared for a crown with shape of premolar teeth, and porcelain crowns were cemented using resin cement (RelyX, 3M, Neuss, Germany). The treatment showed satisfactory aesthetic and functional results at 1-year follow-up. Dental implants were osseo-integrated without marginal bone loss (Figure 10 and Figure 11).

## 3. Discussion

Overall, complex cases require computer-guided surgery to ensure the correct position of dental implants and avoid damage to the neighboring anatomical structures [9]. This case report provides information on the use of digital tools when anatomical structures and aesthetic needs increased the complexity of surgical procedures.

In the first case, both deficient jaw growth and reduced bone volume at the third quadrant hampered the installation of dental implants. Since the patient refused a bone graft, nerve lateralization was required. Nonetheless, installation of dental implants without nerve injury was possible, only due to the accurate treatment planning. This was achieved by the superimposition of bone structure to the planned restoration, which allowed to visualize the optimal position of dental implants [12].

Conversely, the complexity of the second case report relied on the disharmony among neighboring structures. After orthodontic treatment, a wide space of 9 mm hindered proper implant placement planning. Thus, a digital interdisciplinary treatment plan was required to determine the implant position in association with restorative treatments of adjacent teeth.

Dental treatment of patients with ectodermal dysplasia involves a long-term interdisciplinary treatment. Whereas conservative treatments involving orthodontics and removable prosthesis begins at childhood, surgical treatments and fixed restorations are postponed to the adulthood [4]. In this stage, dental implant therapy is recommended to improve patient´s comfort and aesthetic appearance [2,9,10,11,12,13].

Our research group previously showed that people diagnosed with oral abnormalities as manifestations of rare diseases are less satisfied with their oral situation than the general population [14,15]. In this regard, the oral rehabilitation with fixed implant-supported prosthodontics seems to improve the perception about their oral-health quality of life [16]. Although an established treatment protocol of oral manifestations in rare diseases is missing, an individualized treatment aiming the return of acceptable aesthetics and function should be conducted. Patient´s age, their needs, compliance, and physical conditions should be taken in consideration.

Prosthetic-guided implant planning is useful to increase the predictability of treatment outcomes. Currently, it is possible to combine different digital files to the creation of a virtual patient. This is most important for dental implant planning, because it allows taking in consideration functional and aesthetic needs of the patient. However, limitations of the technique not only are related to anatomical conditions, but also to the accuracy of digital files. Inaccuracy of imaging files may influence negatively the final outcome.

Few studies addressed digital planning of implant therapy in patients suffering from oral manifestations of systemic diseases. Nonetheless, Gonzaga et al. (2021) described the facially driven digital planning of a patient diagnosed with ectodermal dysplasia, and showed a satisfactory rehabilitation outcome [9].

A limitation of this case report is the short-term follow-up, which does not enable the drawing of conclusions regarding the post-term outcome. Clinical studies with long-term follow-up should be conducted to establish a treatment protocol to support patients with ectodermal dysplasia requiring dental implant therapy.

## 4. Conclusions

Computer-guided implant placement allowed predictable treatment of complex cases with satisfactory aesthetic and functional results. Adequate surgical and prosthetic planning is considered critical for treatment success.

## Data Availability

Not applicable.
